# A Biofilm Matrix-Associated Protease Inhibitor Protects Pseudomonas aeruginosa from Proteolytic Attack

**DOI:** 10.1128/mBio.00543-18

**Published:** 2018-04-10

**Authors:** Boo Shan Tseng, Courtney Reichhardt, Gennifer E. Merrihew, Sophia A. Araujo-Hernandez, Joe J. Harrison, Michael J. MacCoss, Matthew R. Parsek

**Affiliations:** a Department of Microbiology, University of Washington, Seattle, Washington, USA; b School of Life Sciences, University of Nevada Las Vegas, Las Vegas, Nevada, USA; c Department of Genome Sciences, University of Washington, Seattle, Washington, USA; d Department of Biological Sciences, University of Calgary, Calgary, AB, Canada; University of Michigan—Ann Arbor

**Keywords:** *Pseudomonas aeruginosa*, biofilms, extracellular matrix

## Abstract

Pseudomonas aeruginosa produces an extracellular biofilm matrix that consists of nucleic acids, exopolysaccharides, lipid vesicles, and proteins. In general, the protein component of the biofilm matrix is poorly defined and understudied relative to the other major matrix constituents. While matrix proteins have been suggested to provide many functions to the biofilm, only proteins that play a structural role have been characterized thus far. Here we identify proteins enriched in the matrix of P. aeruginosa biofilms. We then focused on a candidate matrix protein, the serine protease inhibitor ecotin (PA2755). This protein is able to inhibit neutrophil elastase, a bactericidal enzyme produced by the host immune system during P. aeruginosa biofilm infections. We show that ecotin binds to the key biofilm matrix exopolysaccharide Psl and that it can inhibit neutrophil elastase when associated with Psl. Finally, we show that ecotin protects both planktonic and biofilm P. aeruginosa cells from neutrophil elastase-mediated killing. This may represent a novel mechanism of protection for biofilms to increase their tolerance against the innate immune response.

## IMPORTANCE

Proteins associated with the extracellular matrix of bacterial aggregates called biofilms have long been suggested to provide many important functions to the community. To date, however, only proteins that provide structural roles have been described, and few matrix-associated proteins have been identified. We developed a method to identify matrix proteins and characterized one. We show that this protein, when associated with the biofilm matrix, can inhibit a bactericidal enzyme produced by the immune system during infection and protect biofilm cells from death induced by the enzyme. This may represent a novel mechanism of protection for biofilms, further increasing their tolerance against the immune response. Together, our results are the first to show a nonstructural function for a confirmed matrix-interacting protein.

## INTRODUCTION

Pseudomonas aeruginosa is an opportunistic pathogen that causes a variety of chronic infections (1). Many of these chronic infections have been linked to the biofilm mode of growth. Such infections are difficult to eradicate because bacteria in biofilms have a higher tolerance against antimicrobial agents than their planktonic counterparts (2). A key feature of biofilm communities is an extracellular matrix, which surrounds the resident bacteria and is composed of extracellular DNA (eDNA), exopolysaccharides, lipid vesicles, and matrix proteins. While the three exopolysaccharides of the P. aeruginosa biofilm matrix (Psl, Pel, and alginate) (3) have been fairly well studied, our knowledge of the matrix proteins and their roles in the community is very limited as such studies are technically challenging.

While global proteomic approaches have been used to study P. aeruginosa biofilms (4), most studies do not distinguish between proteins derived directly from resident cells in the biofilm and proteins found in the extracellular matrix environment. Many of these studies have characterized the proteins in the total biofilm (cellular and matrix proteins), while others have identified the proteins in the matrix once the cells have been removed. Since the latter requires excessive processing of the biofilm community in order to isolate the matrix proteins from the cells, it is likely that some cells lyse during the processing, leading to contamination by cellular proteins.

To date, studies of matrix proteins have focused primarily on the proteins that provide structural support to the biofilm, such as adhesins, nucleoid-associated proteins, and amyloid proteins (5). However, several exciting roles that extend beyond promoting structural integrity for matrix proteins have been proposed (6). Biochemical activities are found in the matrices of environmental biofilms (7), suggesting that biochemically active matrix proteins may be providing important functions for the community. However, examples of nonstructural proteins that are active while bound to the biofilm matrix are essentially lacking in the literature. Interestingly, there is precedence supporting this possibility in eukaryotic biology, where proteins in the basement membrane have been shown to carry out a multitude of functions (8).

In this study, we identified 60 matrix-associated proteins using a noninvasive proteomic approach. We hypothesized that the extracellular matrix selectively retains biochemically active proteins that aid in the protection of the biofilm. We predict that 19 of the 60 proteins have protective functions. We focused on one candidate matrix protein, ecotin (PA2755), a serine protease inhibitor (9). This protein is of interest because of its ability to inhibit neutrophil elastase, an enzyme produced by the innate immune system during P. aeruginosa respiratory infections (10). During biofilm growth, ecotin levels within the extracellular matrix were found to increase over time. We show that ecotin binds to the biofilm exopolysaccharide Psl and that it inhibits neutrophil elastase when bound to a Psl matrix in a cell-free system. Finally, we show that ecotin can protect both planktonic and biofilm P. aeruginosa cells from neutrophil elastase-mediated cell death. Collectively, these results suggest that the P. aeruginosa biofilm matrix binds to and retains specific proteins that remain active in the extracellular environment, thereby protecting the biofilm community.

## RESULTS

### Identification of proteins enriched in the biofilm matrix.

To identify proteins enriched in the biofilm matrix of P. aeruginosa, we modified a protocol used by Absalon and colleagues in Vibrio cholerae (11). Briefly, the extracellular proteins of mature P. aeruginosa PAO1 biofilms were biotinylated prior to biofilm disruption. The biotinylated proteins were then purified and identified using quantitative mass spectrometry. This “extracellular matrix proteome” was compared to the proteome of the total biofilm, in which the cells in the biofilm were lysed prior to the biotinylation of the proteins. In comparison to the extracellular matrix proteome, the “total biofilm proteome” contains both intracellular and extracellular proteins. Therefore, matrix proteins will be higher in abundance in the extracellular matrix proteome than in the total biofilm proteome. For a negative control, this analysis was also performed in the absence of the biotinylation agent for both the extracellular matrix and total biofilm proteomes.

**FIG 1:**
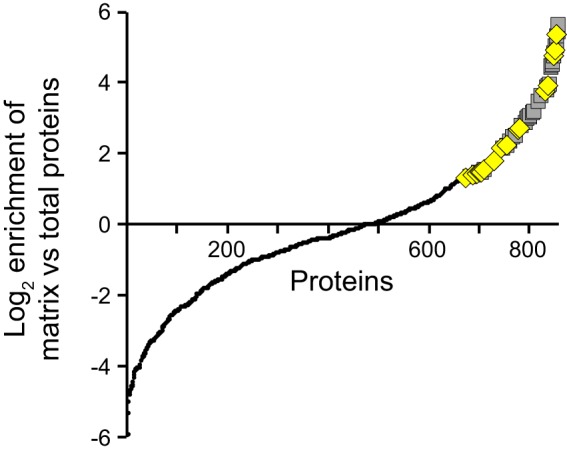
Proteomic analysis of the P. aeruginosa biofilm matrix. Using quantitative mass spectrometry, the extracellular matrix proteome of mature P. aeruginosa biofilms was compared with the total matrix proteome. The spectrum counts of each protein identified in two separate runs of this analysis were compared (857 total proteins; small black circles). The candidate matrix proteins are shown in gray squares (60 total proteins). The yellow diamonds denote proteins that we predict are involved in the protection of the biofilm (19 of the 60 total proteins).

This analysis was conducted twice, and a total of 857 unique proteins were identified in both runs (Fig. 1). The candidate matrix proteins were identified on the basis of the following criteria: (i) the candidate matrix proteins had greater than or equal to two-fold spectrum counts in the extracellular biofilm proteome relative to the total biofilm proteome analysis; (ii) the candidate proteins had at least 10 spectrum counts in the extracellular biofilm proteome; and (iii) the candidate proteins had greater than or equal to two-fold spectrum counts in the biotinylated extracellular biofilm proteome relative to the biofilm proteome without biotinylation. By comparing the proteomes using these high-stringency criteria, we identified 60 proteins that are enriched in the biofilm matrix in both analyses (Fig. 1; see Tables S1 and S2 in the supplemental material). On the basis of their predicted biochemical activities, we categorized 19 of the 60 proteins as potentially playing a role in the protection of the biofilm matrix (Fig. 1 and Table S1). Of these 19 proteins, 12 have predicted oxidoreductase activity based on the InterProScan software package (12), and another four proteins may play a role in redox processes. Of the remaining three proteins predicted to play a role in the protection of the biofilm matrix, two have functions in facilitating protein folding, and one is a serine protease inhibitor, which we chose to further investigate. It is important to note that our classification of these proteins as potentially playing a protective role in the biofilm matrix has not been verified, and it is possible that these proteins provide other functions in the matrix.

10.1128/mBio.00543-18.4 TABLE S1  Proteins enriched in the extracellular biofilm matrix. Download TABLE S1, PDF file, 0.05 MB. Copyright © 2018 Tseng et al. 2018 Tseng et al. This content is distributed under the terms of the Creative Commons Attribution 4.0 International license.

10.1128/mBio.00543-18.5 TABLE S2  Characteristics of proteins enriched in the extracellular biofilm matrix. Download TABLE S2, PDF file, 0.04 MB. Copyright © 2018 Tseng et al. 2018 Tseng et al. This content is distributed under the terms of the Creative Commons Attribution 4.0 International license.

### Ecotin is a candidate matrix protein.

To validate our proteomic analysis, we focused on ecotin, a serine protease inhibitor (9) that is encoded by PA2755 (referred to herein as *eco*). Ecotin was enriched approximately four-fold in the matrix proteome (Table S1). Originally identified in Escherichia coli, ecotin inhibits enzymes, such as trypsin, chymotrypsin, and neutrophil elastase (9). While found in a wide variety of *Gammaproteobacteria*, most bacteria, including P. aeruginosa, that carry an *eco* gene do not produce proteases of the class that ecotin inhibits, which suggests that ecotin does not inhibit a bacterium’s own proteases (13, 14). Instead, it has been suggested that ecotin may protect bacteria from external proteolytic attack in metazoan hosts. Supporting this hypothesis, ecotin in E. coli can protect against neutrophil elastase-mediated cell death *in vitro* (15). Furthermore, ecotin and ecotin-like proteins have been shown to be important for the virulence of Burkholderia pseudomallei (13) and the infectivity of Leishmania major (16). We, therefore, hypothesized that ecotin in the biofilm matrix may protect P. aeruginosa biofilms against proteases such as neutrophil elastase, an enzyme the bacteria commonly come into contact with during chronic biofilm-based infections (10) via the following two mechanisms: (i) ecotin may protect proteins in the matrix from proteolytic degradation by neutrophil elastase, and (ii) ecotin may protect biofilm cells from neutrophil elastase-mediated death. Both possibilities would help preserve biofilm integrity and are not mutually exclusive.

### Ecotin is found to accumulate within the extracellular matrix of mature biofilms.

Since P. aeruginosa has been shown to have strain-to-strain differences in its production of biofilm matrix components (17), we examined ecotin expression in a subset of the sequenced P. aeruginosa strains in the Pseudomonas Genome Database, all of which contain an *eco* gene (18) and are able to produce the P. aeruginosa exopolysaccharides Psl and Pel (17). Via immunoblotting, we found that ecotin is expressed in all nine of the tested clinical and environmental isolates. In stationary phase, the expression levels for these strains were at a level similar to that of our laboratory PAO1 reference strain (Fig. S1).

10.1128/mBio.00543-18.2 FIG S1  Ecotin is expressed in a wide variety of P. aeruginosa isolates. (Top) Ecotin levels in stationary-phase cells of various environmental (environ) and clinical isolates were determined by immunoblotting (IB). (Bottom) An immunoblot for RNA polymerase (IB: RNAP) serves as a loading control. Our wild-type laboratory strain (PAO1) and the isogenic strain lacking ecotin (Δ*eco*) are included as a reference. The environmental or clinical isolates are as follows: plant, strain E2; water-1, MSH3; water-2, MSH10; CF-1 (CF stands for cystic fibrosis), CF18; CF-2, CF127; UTI (urinary tract infection), JJ692; eye, 19660; ear, T56593; blood, X13273. Download FIG S1, PDF file, 0.1 MB. Copyright © 2018 Tseng et al. 2018 Tseng et al. This content is distributed under the terms of the Creative Commons Attribution 4.0 International license.

**FIG 2:**
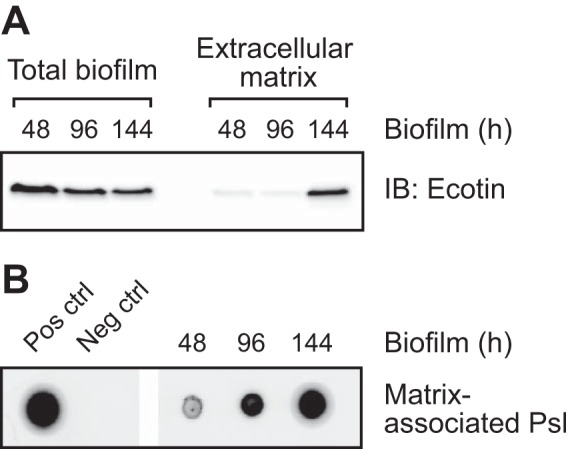
Ecotin accumulates within the extracellular matrix of mature biofilms. Biofilms were prepared similarly to biofilms prepared for the proteomic analysis and harvested at the indicated time points. (A) An immunoblot (IB) of ecotin in the total biofilm and the extracellular matrix at different points in biofilm development. (B) The corresponding immunoblot of the matrix-associated Psl of the samples in panel A. The two leftmost lanes are a positive control (Pos ctrl) and negative control (Neg ctrl).

Next, we determined whether ecotin expression or its presence in the biofilm matrix changes during the course of biofilm growth. Using a culturing methodology similar to that used in the proteomic analysis, we used immunoblotting to test for the presence of ecotin in the total biofilm and the extracellular matrix of biofilms grown for 48, 96, and 144 h. While ecotin is present at approximately equal levels in the total harvested biofilm biomass at all three time points of development, it was found in the extracellular matrix at high levels only in the older 144-h biofilms (Fig. 2A). This increase in ecotin in the extracellular matrix was associated with a corresponding increase in the amounts of matrix-associated Psl exopolysaccharide (Fig. 2B).

### Ecotin binds to the biofilm exopolysaccharide Psl.

**FIG 3:**
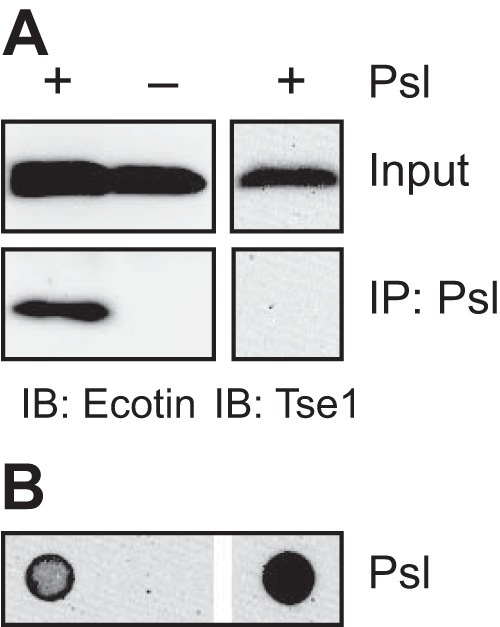
Ecotin binds to the exopolysaccharide Psl. Psl was immunoprecipitated from cell-free supernatants of stationary-phase cultures with anti-Psl antibodies, and coprecipitating proteins were eluted and detected by immunoblotting. For ecotin, the supernatants were from strains overexpressing ecotin in wild-type (+) or *psl* mutant (−) backgrounds. For Tse1, the supernatant was from a Δ*retS* mutant strain. Input and Psl immunoprecipitation (IP: Psl) are shown. (A) Immunoblots (IB) for ecotin and Tse1. (B) The corresponding Psl immunoblot of the samples in panel A.

Because the accumulation of ecotin in the matrix was temporally associated with the accumulation of Psl (Fig. 2), we speculated that ecotin interacts with Psl. To test this hypothesis, we used a coimmunoprecipitation method previously described to identify CdrA as a Psl-binding protein (19). Using anti-Psl antibody-coated beads, we precipitated Psl and any interacting proteins from cell-free supernatants of *eco*-overexpressing strains with wild-type or *psl* mutant backgrounds. Ecotin was then detected by immunoblotting. Ecotin coprecipitated with anti-Psl antibodies in the presence, but not in the absence, of Psl (Fig. 3). For a negative control, we examined whether another protein when overexpressed would coimmunoprecipitate with Psl. While Tse1, an effector of the type VI secretion system (20), was expressed, it did not precipitate with anti-Psl antibodies even in the presence of Psl (Fig. 3). In addition, using the proteomic analysis that identified ecotin as a matrix-interacting protein, we failed to identify ecotin as a protein enriched in the matrix of a biofilm lacking Psl. In the two separate runs, only nine and two spectral counts were obtained for ecotin in the biotinylated extracellular biofilm proteome of a Psl-negative biofilm. Since these counts are below our threshold of 10 counts, ecotin was eliminated from further analysis of matrix-enriched proteins for the Psl-negative biofilm. Together, these results confirm that ecotin binds to the biofilm matrix via the exopolysaccharide Psl and validates our screening methodology for matrix proteins.

### Matrix-associated ecotin can inhibit neutrophil elastase activity.

**FIG 4:**
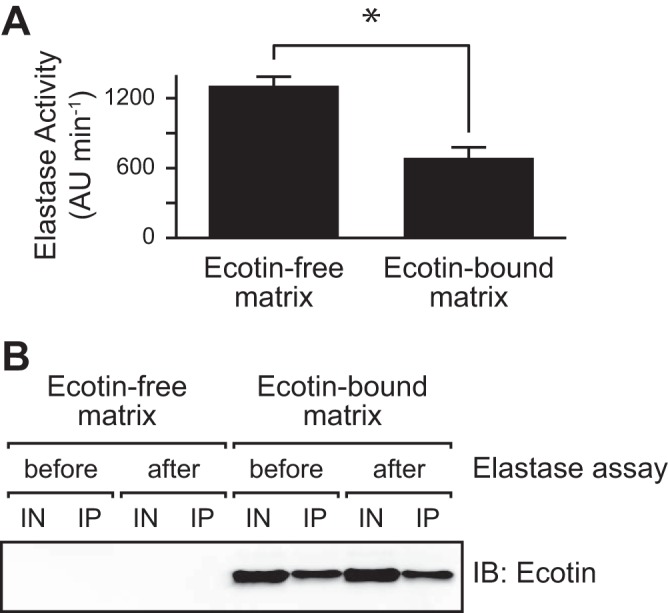
Matrix-bound ecotin inhibits neutrophil elastase. Psl was immunoprecipitated (IP) from supernatants obtained from cells overexpressing either Psl alone (Δ*eco*; P_*BAD*_-*psl*) or both Psl and ecotin (Δ*eco*; P_*BAD*_-*psl*; P_*BAD*_-*eco*). Human neutrophil elastase and a fluorogenic substrate were added. Enzymatic activity was monitored via cleavage of the substrate. (A) Elastase activity in the presence of ecotin-free and ecotin-bound matrix material. Elastase activity is shown in the change in arbitrary units (ΔAU) per change in time (in minutes). The error bars show 1 standard error of the mean (SEM) (*n* = 5). Values that are significantly different (*P* < 0.01) by Student’s *t* test are indicated by the bar and asterisk. (B) Immunoblot of ecotin before and after the elastase activity assay in panel A. Samples contained either ecotin-free matrix or ecotin-bound matrix. IN, input for immunoprecipitation; IP, immunoprecipitated matrix material.

Since a protein may lose its functionality outside the cell when interacting with the matrix, we sought to determine whether matrix-bound ecotin is functionally active in a cell-free system. To obtain matrix-bound ecotin, we immunoprecipitated Psl from cell-free supernatants of cells that were overproducing both ecotin and Psl. As a control, Psl was immunoprecipitated from cell-free supernatants of Δ*eco* cells that were overproducing only Psl. The activity of neutrophil elastase was then tested in the presence of ecotin-bound or ecotin-free matrix material. With the ecotin-bound matrix, neutrophil elastase activity was inhibited 52.1% ± 10.2% (mean ± standard deviation [SD]; *n* = 5; *P* < 0.01) compared to that with the ecotin-free matrix (Fig. 4A). This partial inhibition is likely due to experimental technicalities: the immunoprecipitated ecotin-bound matrix contained only 16.5 ± 3.2 nM ecotin (*n* = 3) compared to the 100 nM neutrophil elastase added to the experiment. Furthermore, this inhibition is likely not due to the release of ecotin from the matrix, since approximately equal amounts of ecotin were bound to the matrix before and after the elastase activity assay by immunoblotting (Fig. 4B). Nonetheless, these results show that matrix-associated ecotin can inhibit neutrophil elastase, suggesting that ecotin in the biofilm matrix can protect other matrix proteins from proteolytic degradation.

### Ecotin can protect planktonic P. aeruginosa from neutrophil elastase-mediated killing.

**FIG 5:**
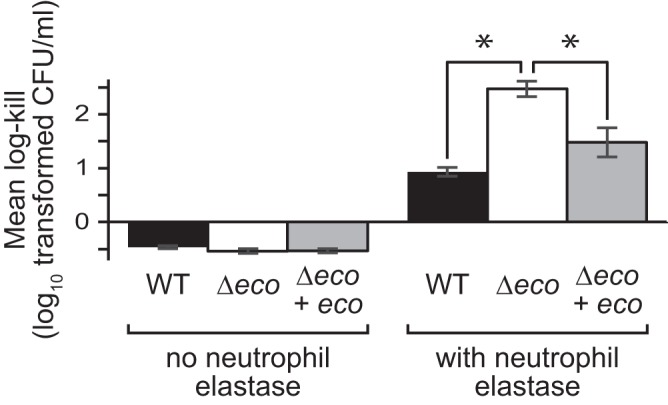
Ecotin protects planktonic P. aeruginosa cells from neutrophil elastase-mediated killing. Planktonic cells were grown to stationary phase (OD_600 _of ~2.0) and then treated with neutrophil elastase for 2 h before cell viability was determined for wild-type (WT) cells, cells lacking ecotin (Δ*eco*), and Δ*eco* cells complemented ectopically with ecotin (Δ*eco* + *eco*). Log-transformed CFU/ml after treatment was subtracted from the value before treatment to obtain the log kill. Positive numbers represent cell death, and negative numbers represent cell growth. Error bars show 1 SEM (*n* = 3). Values that are significantly different (*P* < 0.01) by analysis of variance (ANOVA) with a *posthoc* Tukey test are indicated by a bar and asterisk.

Since ecotin has previously been shown to protect planktonic E. coli from neutrophil elastase-mediated killing (15), we looked at whether ecotin could protect P. aeruginosa planktonic and biofilm cells from neutrophil elastase. Using planktonic cells, we compared the effect of neutrophil elastase treatment on the viability of wild-type versus Δ*eco* cells (Fig. 5). While wild-type cells decreased in viability when treated with neutrophil elastase (a decrease of 0.93 ± 0.14 log_10_-transformed CFU/ml [mean ± SD]; *n* = 3), Δ*eco* cells had significantly more cell death (a loss of 2.47 ± 0.25 log_10_-transformed CFU/ml; *n* = 3; *P* < 0.01). This difference in viability is due to neutrophil elastase, since wild-type and Δ*eco* cells grew to similar levels in the absence of neutrophil elastase (Fig. 5). Furthermore, this defect in Δ*eco* cells could be rescued by complementing the strain with an ectopic copy of *eco* driven under its native promoter. When treated with neutrophil elastase, the complemented strain had a decrease in viability that was indistinguishable from that of wild-type cells (a decrease of 1.48 ± 0.47 log_10_-transformed CFU/ml; *n* = 3; *P* = 0.10).

### Ecotin can protect P. aeruginosa in a biofilm from neutrophil elastase-mediated killing.

**FIG 6:**
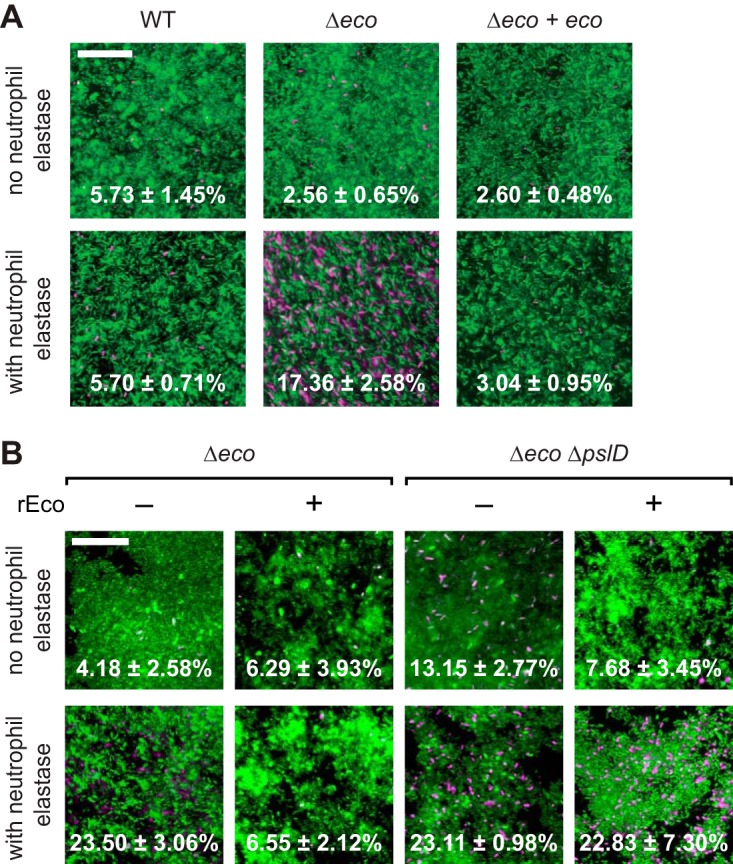
Ecotin protects P. aeruginosa biofilm cells from neutrophil elastase-mediated killing. (A) Static biofilms of the wild-type (WT) strain, Δ*eco* mutant, and Δ*eco* mutant complemented with ecotin (Δ*eco* + *eco*) were grown on glass slides for 20 h and then treated with neutrophil elastase for 30 m. (B) The indicated biofilms were exposed to recombinant ecotin (rEco) for 15 min and then rinsed before elastase treatment. The adherent biomass was then stained with Syto9 to visualize the living cells (green) and propidium iodide to visualize the dead cells (magenta). Representative images are shown with the percentage of cells that are dead ± 1 SEM (*n* = 3). Bars, 25 µm.

While our data (Fig. 4 and 5) support our hypothesis that ecotin in the biofilm matrix can protect the biofilm against neutrophil elastase, to more directly test this hypothesis, we treated static glass-grown biofilms with neutrophil elastase and determined the percentage of cells killed by the treatment based on viability (propidium iodide) staining. As expected, we saw that indeed Δ*eco* biofilms were more susceptible to killing by neutrophil elastase than wild-type biofilms were (Fig. 6A). In the absence of neutrophil elastase treatment, Δ*eco* biofilms had an equivalent number of dead cells as that in wild-type biofilms (*P* = 0.57; *n* = 3). In comparison, after neutrophil elastase treatment, Δ*eco* biofilms had significantly more cell death than wild-type biofilms did (*P* < 0.01; *n* = 3). Furthermore, complementation of *eco* rescued the biofilm susceptibility defect (*P* = 0.70; *n* = 3). These results show that ecotin can protect the biofilm from neutrophil elastase-mediated killing. This experiment, however, does not separate the contribution of the cellular ecotin in the periplasm from that of the extracellular matrix-bound ecotin.

To isolate the contribution of the extracellular matrix-bound ecotin, we examined whether exogenous addition of ecotin can protect Δ*eco* biofilms from neutrophil elastase-mediated cell death (Fig. 6B). We first purified recombinant ecotin and confirmed that this protein was capable of inhibiting neutrophil elastase *in vitro* (Fig. S2), similar to that previously seen by Eggers and colleagues (15). We then tested the ability of this protein to rescue neutrophil elastase-mediated killing of the Δ*eco* biofilm by exposing the biofilms to this recombinant ecotin and then rinsing the biofilms to remove any ecotin that did not bind to the matrix before neutrophil elastase treatment. After neutrophil elastase treatment, Δ*eco* biofilms exposed to recombinant ecotin had significantly fewer dead cells compared to Δ*eco* biofilms not exposed to recombinant ecotin (*P* < 0.05; *n* = 3) and had an equivalent number of dead cells to that of Δ*eco* biofilms that were not treated with neutrophil elastase (*P* = 0.90; *n =* 3). This rescue of neutrophil elastase-mediated cell death in Δ*eco* biofilms via the addition of recombinant ecotin is dependent on Psl. Unlike Δ*eco* biofilms, biofilms of a mutant strain that lack both ecotin and Psl production (Δ*eco* Δ*pslD* mutant) that were exposed to recombinant ecotin had significantly more cell death after neutrophil elastase treatment compared to biofilms not exposed to neutrophil elastase (*P* < 0.05; *n* = 3). Therefore, Psl is required for the supplemented ecotin to protect the biofilm against neutrophil elastase-mediated cell death. This requirement for Psl is likely only for the extracellular portion, and not the periplasmic cellular portion, of ecotin in wild-type P. aeruginosa biofilms, since endogenous cellular ecotin in other bacteria protects against neutrophil elastase independent of Psl. These results further support our conclusion that ecotin is a Psl-interacting protein in the biofilm matrix and that matrix-associated ecotin is functional. Together, our results show that ecotin in the matrix can protect not only other matrix proteins but also biofilm cells from proteolytic attack.

10.1128/mBio.00543-18.3 FIG S2  Recombinant P. aeruginosa ecotin can inhibit neutrophil elastase *in vitro*. (A) Coomassie blue-stained gel of purified recombinant P. aeruginosa ecotin. (B) *In vitro* assay of neutrophil elastase activity using a fluorogenic substrate. Neutrophil elastase activity without recombinant ecotin (black squares) and with two-fold molar excess of recombinant ecotin (gray diamonds) are shown. Error bars represent 1 standard deviation (SD). Download FIG S2, PDF file, 0.1 MB. Copyright © 2018 Tseng et al. 2018 Tseng et al. This content is distributed under the terms of the Creative Commons Attribution 4.0 International license.

## DISCUSSION

While matrix proteins have been suggested to provide several nonstructural functions to the biofilm community (6), examples of such proteins in the literature are very limited. We, therefore, undertook a proteomic approach to identify matrix proteins in P. aeruginosa biofilms (Fig. 1; also see Table S1 in the supplemental material). As a proof of principle, we characterized one candidate matrix protein, ecotin. This protein was of interest because it inhibits neutrophil elastase, a bactericidal enzyme produced by the innate immune system during P. aeruginosa respiratory infections. Our data strongly suggest that this matrix protein is active when bound to the exopolysaccharide Psl (Fig. 4A and 6), and our data show that it can protect P. aeruginosa in a biofilm from neutrophil elastase-mediated killing (Fig. 6). While recombinant ecotin has been previously shown to inhibit neutrophil elastase (15), our data strongly suggest that not only does the endogenous P. aeruginosa ecotin inhibit neutrophil elastase but that it does so when bound to the biofilm matrix. This is not an inherently obvious result, as binding to the matrix, or any other molecule, could have impaired ecotin function. We propose that matrix-associated proteins play a crucial role in biofilm-mediated resistance to host defenses.

The major advantage of our proteomic approach is its selectivity. First, biofilms were grown under continuous flow. Since cell lysis is a natural part of biofilm formation, the flow allows proteins that do not interact with the matrix to be continuously washed away. This flow-based growth method is, therefore, more stringent for identifying matrix-interacting proteins than the static methods for biofilm growth that have been used in the past, in which a limited number of washes are used to remove noninteracting proteins (21–23). Second, for the extracellular matrix proteome, the proteins in the biofilm matrix are labeled prior to mechanical/physical disruption of the system, limiting the effects of experimentally introduced cell lysis and greatly reducing the probability of cellular protein contamination. Third, the extracellular matrix proteome is compared to the total biofilm proteome at the same point in biofilm development, eliminating the effects of general proteome changes that are known to occur over biofilm development (24). It should be noted, however, that we have confirmed only one protein, ecotin, in our list of candidates (Table S1) as a bona fide matrix-interacting protein. While outside the scope of this work, the other candidates still need to be individually verified.

Our list of 60 candidate matrix proteins is likely far from exhaustive. As a control, abundant intracellular proteins that are not expected to be in the matrix, such as subunits of RNA polymerase, were identified in the total biofilm proteome, but not in the extracellular matrix proteome. However, due to the high-stringency criteria and comparison approach used, the number of false-negative results is likely high. For instance, proteins that are high in abundance in the cell, such as nucleoid-associated proteins (25), or that are identified in the sample without the biotinylation agent, such as flagellin, were eliminated as potential candidates. Furthermore, our purification method likely selected against proteins in outer membrane vesicles (OMVs), as we saw only five such proteins of the 60 proteins identified (Table S2). These results are in contrast to previously published work for P. aeruginosa (21, 23), in which larger percentages of outer membrane proteins were identified. However, the many differences in the methodologies used likely explain this discrepancy. For instance, since our study involved more purification steps, outer membrane-associated proteins, especially adhesins such as LecA (26), LecB (27), and CdrA (19), may have been selected against, hence the lower percentage of these proteins in our data set.

A majority of the candidate matrix proteins we identified, including ecotin, are not predicted to be exported outside the cell (Table S2). It is unclear whether these proteins become extracellular through nonclassical protein secretion mechanisms (28) or via cell lysis, which is a natural part of biofilm formation (29). Independently of how these proteins become extracellular, we hypothesize that matrix proteins are selectively retained by the biofilm via their interaction with structural matrix polymers (e.g., eDNA, polysaccharides, and amyloid proteins) and that non-matrix-interacting proteins are lost to the environment via diffusion. Supporting this hypothesis, our data show that ecotin binds to Psl both in a cell-free system (Fig. 3A) and in the biofilm (Fig. 6B). Furthermore, we would expect that the presence of any specific matrix protein should temporally correlate with the presence of its interacting structural matrix component, which we also see for ecotin and Psl (Fig. 2).

While very different fields, there are parallels between the basement membrane of metazoans and the bacterial extracellular biofilm matrix. Similar to the biofilm matrix, the basement membrane was once considered to be simply a passive scaffold for polarized epithelial cell attachment, but the basement membrane has become a major area of study due to its many roles and associations with human disease (8). While both matrices clearly provide structural support to the cells interacting with them, the interaction of cells with specific components of both matrices also leads to complex signaling within the cells (30, 31). While speculative, it is tempting to draw other parallels. For instance, enzymes within the basement membrane that remodel the matrix are important for proper tissue development (32, 33). This may also be true for biofilms and the predicted enzymatic matrix proteins that we identified. Additionally, the basement membrane contains matrix components that when processed can protect the host (34–36), similar to how ecotin and other matrix proteins may be protecting the biofilm.

Components of the biofilm matrix are known to protect the resident bacteria against the host innate immune response (37). For instance, eDNA increases the resistance of P. aeruginosa against the cationic antimicrobial peptides secreted by leukocytes (38), and the P. aeruginosa exopolysaccharides alginate and Psl inhibit phagocytosis by neutrophils and macrophages (39–41), as well as limit complement activation (41, 42). Adding to the repertoire of the protective mechanisms in the biofilm, our results suggest that ecotin in the biofilm matrix can protect matrix proteins and the resident cells from proteolytic attack. This is a novel mechanism by which the biofilm may protect itself against a key mediator of the host innate immune response. Our proteomic analysis also identified multiple proteins with oxidoreductase activity, suggesting that there may be matrix proteins that can mitigate the stress induced by the neutrophil oxidative burst in the extracellular space of biofilms. Therefore, the protective effects of P. aeruginosa biofilms against the innate immune response via its matrix are potentially multifaceted and likely more complicated than currently envisioned.

## MATERIALS AND METHODS

Full, detailed descriptions of all the methods used are described in Text S1 in the supplemental material.

10.1128/mBio.00543-18.1 TEXT S1  Supplemental methods and references. Download TEXT S1, PDF file, 0.1 MB. Copyright © 2018 Tseng et al. 2018 Tseng et al. This content is distributed under the terms of the Creative Commons Attribution 4.0 International license.

### Bacterial strains, growth conditions, and antibiotics.

The bacterial strains, plasmids, and primers used in this study are listed in Tables S3 to S5, respectively. Construction of the strains used in this study are described in Text S1. Unless otherwise noted, bacteria were grown at 37°C in lysogeny broth (LB) or in Vogel-Bonner minimal medium (43).

10.1128/mBio.00543-18.6 TABLE S3  Bacterial strains used in this study. Download TABLE S3, PDF file, 0.04 MB. Copyright © 2018 Tseng et al. 2018 Tseng et al. This content is distributed under the terms of the Creative Commons Attribution 4.0 International license.

10.1128/mBio.00543-18.7 TABLE S4  Plasmids used in this study. Download TABLE S4, PDF file, 0.03 MB. Copyright © 2018 Tseng et al. 2018 Tseng et al. This content is distributed under the terms of the Creative Commons Attribution 4.0 International license.

10.1128/mBio.00543-18.8 TABLE S5  Oligonucleotides used in this study. Download TABLE S5, PDF file, 0.04 MB. Copyright © 2018 Tseng et al. 2018 Tseng et al. This content is distributed under the terms of the Creative Commons Attribution 4.0 International license.

### Antibodies and immunoblots.

Primary antibodies were diluted in 5% milk in phosphate-buffered saline (PBS) with 0.05% Tween 20: anti-ecotin sera (raised against AKLDEKVPYPKADC; Covance) diluted 1/400, anti-Psl antibodies (MedImmune) (44) diluted 1/3,000, and anti-Tse1 antibodies (20) diluted 1/2,000. Psl immunoblotting was performed as previously described (45). For SDS-PAGE, proteins were separated on an 18% Tris HCl gel and transferred to nitrocellulose for immunoblotting.

### Identification of proteins enriched in the matrix.

Tube biofilms were grown as previously described (46) in amine-free medium [60 mM trisodium citrate, 15 mM K_2_HPO_4_, 50 mM (NH_4_)_2_SO_4_, 1.33 mM MgSO_4_, 140 µM CaCl_2_, 8.5 µM ZnSO_4_, 3.9 µM FeSO_4_, pH 7.5] for 6 days at 25°C. For the total biofilm proteome analysis, the cells in the biofilm biomass were first lysed before the proteins in the sample were biotinylated. For the extracellular biofilm proteome, the extracellular proteins in the biofilm were biotinylated before the biomass was collected and the cells were removed from the matrix material. The biotinylated proteins were purified and digested with trypsin. The resulting peptides were then identified by liquid chromatography coupled to tandem mass spectrometry (LC-MS/MS).

### Psl coimmunoprecipitation.

Psl coimmunoprecipitations were performed as previously described with the following modifications (19). Briefly, anti-Psl antibodies (MedImmune)-conjugated magnetic protein A Dynabeads (Life Technologies) were incubated with cell-free supernatants from stationary-phase cultures. Proteins coprecipitating with Psl were eluted with Laemmli buffer and analyzed by immunoblotting.

### Ecotin expression profiling.

For planktonic expression experiments, cells were resuspended in Laemmli buffer at 10^10^ CFU/ml and analyzed by immunoblotting. To determine when ecotin is associated with the biofilm matrix, tube biofilms were grown as described above to the desired time point. Half of the sample was lyophilized to obtain the dry weight. In the other half of the sample, the cells were removed from the matrix. The proteins in the supernatant were normalized based on their dry weight measurements and analyzed by SDS-PAGE.

### Neutrophil elastase inhibition assay.

Psl was precipitated on the magnetic beads as described above, and neutrophil elastase (Millipore) at 100 nM and the fluorogenic substrate (MeOSucAAPV-AMC; Millipore) at 0.25 mM were then added. Cleavage of the substrate was measured at an excitation wavelength of 380 nm and an emission wavelength of 460 nm (Ex380/Em460). After the experiment, the beads were recovered, and the proteins were analyzed by SDS-PAGE as described above.

### Neutrophil elastase killing assay.

For planktonic experiments, cells at an OD_600_ of ~2.0 were treated with neutrophil elastase (Oxford Biomedical Research) at 250 µg/ml at 37°C for 2 h before viable-cell counts were determined. For biofilm experiments, biofilms grown for 20 h on glass slides were treated with 100 µM ecotin and 2 μM neutrophil elastase (Millipore) for 30 min. The biomass was stained with 5 μM Syto9 (Molecular Probes) and 30 μM propidium iodide (Sigma-Aldrich) for 15 min before imaging with a Zeiss LSM 510 confocal laser scanning microscope (Carl Zeiss).
