# Supplementary material for: A Biofilm Matrix-Associated Protease Inhibitor Protects Pseudomonas aeruginosa from Proteolytic Attack
Source: mBio. 2018 Apr 10;9(2):e00543-18. doi: 10.1128/mBio.00543-18 (PMC5893882; doi:10.1128/mBio.00543-18)
Supplement: TABLE S1 [file mbo001183821st1.pdf]

Supplementary Table S1. Proteins enriched in the extracellular biofilm matrix.

PA#	Name[a]	% Coverage in Matrix with Biotin Sample		Spectral Counts (SC) in Matrix with Biotin Sample		Ratio of SC Matrix with vs without Biotin Samples		Ratio of SC Matrix vs Total Samples	
		Run 1	Run 2	Run 1	Run 2	Run 1	Run 2	Run 1	Run 2
PA2991	sth; soluble pyridine nucleotide transhydrogenase	40.73	45.47	47	54	NA	NA	NA	NA
PA4561	ribF; riboflavin kinase	28.21	28.85	54	33	NA	NA	NA	NA
PA3896	probable 2-hydroxyacid dehydrogenase	27.69	42.77	47	35	NA	NA	NA	NA
PA4872	oxaloacetate decarboxylase	34.84	38.33	37	29	NA	NA	NA	NA
PA5516	pdxY; pyridoxamine kinase	22.92	30.21	33	28	NA	NA	NA	NA
PA3356	pauA5; Glutamylpolyamine synthetase	33.9	23.73	14	43	NA	NA	NA	NA
PA4035	hypothetical protein	16.22	52.55	44	10	NA	NA	NA	NA
PA3582	glpK; Glycerokinase	20.59	21.58	23	20	NA	NA	NA	NA
PA3689	yhdM; probable transcriptional regulator	21.79	44.23	20	11	NA	NA	NA	NA
PA4657	hypothetical protein	24.16	23.85	15	15	NA	NA	NA	NA
PA2903	cobJ; precorrin-3 methylase	18.78	12.34	11	18	NA	NA	NA	NA
PA0593	pdxA; pyridoxal phosphate biosynthetic protein	40.59	45.54	13	14	NA	NA	NA	NA
PA0857	bolA; morphogene protein	35.34	46.64	11	11	NA	NA	NA	NA
PA0402	pyrB; aspartate carbamoyltransferase	16.69	39.25	113	270	NA	54.00	12.56	67.50
PA1927	metE; cobalamin-independent methionine synthase	26.63	18.15	17	142	NA	17.75	NA	28.40
PA4676	yadF; probable carbonic anhydrase	28.37	43.26	55	26	NA	NA	27.50	NA
PA2081	kynB; kynurenine formamidase	74.65	68.08	51	41	NA	NA	25.50	NA
PA2491	mexS	39.53	51.92	66	50	22.00	8.33	5.50	25.00
PA0401	pyrX; noncatalytic dihydroorotase-like protein	82.63	87.13	79	132	NA	66.00	11.29	18.86
PA3636	kdsA; Kdo-8-P synthase	35.94	43.77	74	34	NA	NA	14.80	NA
PA2445	gcvP2; glycine decarboxylase	18.35	36.6	87	47	NA	NA	14.50	NA
PA4919	pncB1; nicotinate phosphoribosyl-transferase	19.05	34.09	60	30	NA	NA	20.00	7.50

PA3028	moeA2; molybdopterin biosynthetic protein	31.85	39.01	39	32	NA	NA	13.00	NA
<u>PA4022</u>	hdhA; hydrazone dehydrogenase	9.88	33.6	22	16	NA	NA	11.00	NA
PA3001	probable glyceraldehyde 3-phosphate dehydrogenase	53.58	41	173	281	21.63	93.67	4.22	14.05
PA5143	hisB; imidazoleglycerol-phosphate dehydratase	40.61	40.61	102	70	34.00	NA	7.85	8.75
PA3029	moaB2; molybdopterin biosynthetic protein	78.77	83.8	141	136	47.00	45.33	7.42	9.07
PA1777	oprF; major porin and structural outer membrane porin	33.14	41.14	258	125	12.29	20.83	8.06	8.33
PA3613	hypothetical protein	31.84	44.69	90	103	3.10	2.71	6.00	10.30
PA0548	tktA; transketolase	21.34	30.49	80	29	26.67	NA	10.00	4.83
PA4370	icmP; Insulin-cleaving metalloproteinase	21.75	34.08	91	26	3.64	26.00	5.06	8.67
PA0353	ilvD; dihydroxy-acid dehydratase	54.37	55.56	79	46	2.08	2.56	9.88	3.83
<u>PA5312</u>	pauC; Aldehyde dehydrogenase	56.14	47.28	57	137	7.13	45.67	2.48	10.54
<u>PA1049</u>	pdxH; pyridoxine 5'-phosphate oxidase	35.85	88.68	24	23	NA	NA	8.00	4.60
PA3814	iscS; L-cysteine desulfurase	31.19	32.67	52	49	2.17	4.45	7.43	4.08
PA4376	pncB2; nicotinate phosphoribosyl-transferase	13.82	18.09	37	23	NA	NA	NA	5.75
PA4602	glyA3; serine hydroxyl-methyltransferase	44.12	49.4	169	139	5.63	3.48	4.83	6.62
PA2634	aceA; isocitrate lyase	44.07	38.42	68	134	22.67	44.67	3.09	7.88
PA5343	hypothetical protein	15.55	35.34	30	13	2.73	3.25	6.00	4.33
PA4778	cueR	26.52	38.64	20	10	10.00	NA	NA	5.00
<u>PA5018</u>	msrA; peptide methionine sulfoxide reductase	46.05	48.37	105	74	5.00	8.22	3.18	6.17
PA4848	accC; acetyl-CoA carboxylase	49.22	56.79	110	103	3.55	4.48	4.40	4.90
PA1123	hypothetical protein	40.76	51.63	32	13	4.00	NA	2.46	6.50
<u>PA2755</u>	eco; ecotin precursor	62.18	62.18	40	41	8.00	NA	3.64	5.13
PA3085	hypothetical protein	43.68	70.11	32	14	8.00	NA	4.00	NA
PA0070	tagQ1	24.67	36.51	40	22	NA	22.00	4.44	2.44
<u>PA4759</u>	dapB; dihydro-dipicolinate reductase	57.84	73.88	141	96	6.71	13.71	3.13	3.69
PA2849	ohrR	15.23	22.52	21	12	3.50	2.00	3.50	2.40
<u>PA2640</u>	nuoE; NADH dehydrogenase I chain E	19.28	36.75	29	10	4.14	5.00	2.90	NA
<u>PA3736</u>	hom; homoserine dehydrogenase	14.75	16.36	12	14	NA	NA	3.00	2.80
PA4450	murA; enoylpyruvate transferase	16.39	19	18	10	2.00	2.00	3.60	2.00

PA1287	probable glutathione peroxidase	31.98	41.28	44	17	14.67	NA	3.14	2.43
PA0263/ PA1512/ PA5267	hcpA, B, C; secreted protein Hcp	27.61	31.86	47	31	5.88	3.88	3.36	2.21
PA3480	dcd; dCTP deaminase	24.47	40.43	17	13	NA	NA	3.40	2.17
PA2966	acpP; acyl carrier protein	37.18	46.15	177	176	4.92	6.52	2.64	2.89
PA2119	adh; alcohol dehydrogenase	9.84	22.4	23	13	2.88	2.60	3.29	2.17
PA4176	ppiC2; peptidyl-prolyl cis-trans isomerase	92.47	92.47	222	148	6.53	9.87	2.61	2.60
PA3046	hypothetical protein	72.81	73.68	110	47	9.17	5.22	2.44	2.61
PA0195	pntAA; putative NAD (P) transhydrogenase	15.86	35.22	30	22	NA	NA	3.00	2.00
PA4443	cysD; sulfate adenylate transferase subunit 2	26.23	30.49	14	20	3.50	4.00	NA	2.00

[a] Names based on nomenclature in the Pseudomonas Genome Database.
NA, could not be calculated due to a lack of spectral counts in the extracellular matrix without biotin sample or the total biofilm sample.
Underline, predicted to have protective function in the biofilm matrix.
