# Supplementary material for: A Biofilm Matrix-Associated Protease Inhibitor Protects Pseudomonas aeruginosa from Proteolytic Attack
Source: mBio. 2018 Apr 10;9(2):e00543-18. doi: 10.1128/mBio.00543-18 (PMC5893882; doi:10.1128/mBio.00543-18)
Supplement: TABLE S2 [file mbo001183821st2.pdf]

Supplementary Table S2. Characteristics of proteins enriched in the extracellular biofilm matrix.

PA#	Name[a]	Molecular Weight (kD)[b]	Isoelectric Point (pH)[b]	Hydropathicity (GRAVY score)[b]	Subcellular localization[c]
PA0070	tagQ1	31.7	8.93	-0.481	Outer membrane vesicle (class 1)
PA0195	pntAA; putative NAD (P) transhydrogenase	38.6	5.93	0.154	Cytoplasmic membrane (class 2,3)
PA0263/ PA1512/ PA5267	hcpA, B, C; secreted protein Hcp	19.1	5.76	-0.449	Extracellular (class 3)
PA0353	ilvD; dihydroxy-acid dehydratase	65.2	5.98	-0.11	Cytoplasmic (class 3)
PA0401	pyrX; noncatalytic dihydroorotase-like protein	44.1	5.75	0.103	Cytoplasmic (class 3)
PA0402	pyrB; aspartate carbamoyltransferase	36.6	6.8	-0.114	Cytoplasmic (class 2,3)
PA0548	tktA; transketolase	72.2	4.97	-0.212	Cytoplasmic (class 3)
PA0593	pdxA; pyridoxal phosphate biosynthetic protein	34.9	6.62	0.121	Cytoplasmic (class 3)
PA0857	bolA; morphogene protein	11.2	7.3	-0.308	Unknown (class 3)
PA1049	pdxH; pyridoxine 5'-phosphate oxidase	24.9	6.8	-0.726	Cytoplasmic (class 3)
PA1123	hypothetical protein	12.2	4.65	-0.327	Unknown (class 3)
PA1287	probable glutathione peroxidase	20.2	8.3	-0.294	Periplasmic (class 3)
PA1777	oprF; major porin and structural outer membrane porin	37.6	4.75	-0.443	Outer membrane (class 1)
PA1927	metE; cobalamin-independent methionine synthase	86.2	5.67	-0.227	Cytoplasmic (class 3)
PA2081	kynB; kynurenine formamidase	23.2	5.21	-0.06	Cytoplasmic (class 3)
PA2119	adh; alcohol dehydrogenase	38.6	5.4	0.099	Cytoplasmic (class 3)
PA2445	gcvP2; glycine decarboxylase	103.9	5.97	-0.101	Cytoplasmic (class 3)
PA2491	mexS	36.8	5.26	0.055	Cytoplasmic (class 3)
PA2634	aceA; isocitrate lyase	58.9	5.91	-0.317	Cytoplasmic (class 2,3)
PA2640	nuoE; NADH dehydrogenase I chain E	18.1	4.82	-0.072	Cytoplasmic (class 3)
PA2755	eco; ecotin precursor	17.2	6.52	-0.206	Periplasmic (class 2,3)
PA2849	ohrR	16.8	5.26	-0.059	Cytoplasmic (class 3)

PA2903	cobJ; precorrin-3 methylase	58.9	5.94	0.125	Cytoplasmic (class 3)
PA2966	acpP; acyl carrier protein	8.7	3.79	-0.301	Cytoplasmic (class 3)
PA2991	sth; soluble pyridine nucleotide transhydrogenase	51.2	6.68	-0.146	Cytoplasmic (class 2,3)
PA3001	probable glyceraldehyde-3-phosphate dehydrogenase	50.1	8.04	-0.168	Outer membrane vesicle (class 1)
PA3028	moeA2; molybdopterin biosynthetic protein	43.4	4.96	-0.001	Cytoplasmic (class 3)
PA3029	moaB2; molybdopterin biosynthetic protein	19.2	5.62	0.031	Cytoplasmic (class 3)
PA3046	hypothetical protein	13	4.5	-0.256	Unknown (class 3)
PA3085	hypothetical protein	10	4.59	-0.603	Unknown (class 3)
PA3356	pauA5; Glutamylpolyamine synthetase	45.2	5.56	-0.109	Cytoplasmic (class 3)
PA3480	dcd; dCTP deaminase	21.2	6.26	-0.252	Cytoplasmic (class 3)
PA3582	glpK; Glycerokinase	56	5.87	-0.296	Cytoplasmic (class 3)
PA3613	hypothetical protein	88.4	6.38	-0.282	Cytoplasmic (class 3)
PA3636	kdsA; Kdo-8-P synthase	31.1	6.51	-0.033	Cytoplasmic (class 1,3)
PA3689	yhdM; probable transcriptional regulator	17.7	6.78	-0.559	Cytoplasmic (class 3)
PA3736	hom; homoserine dehydrogenase	46.2	5.09	0.135	Cytoplasmic (class 3)
PA3814	iscS; L-cysteine desulfurase	44.7	5.92	-0.253	Cytoplasmic (class 3)
PA3896	probable 2-hydroxyacid dehydrogenase	35.6	8.23	-0.109	Cytoplasmic (class 2,3)
PA4022	hdhA; hydrazone dehydrogenase	55	5.49	-0.079	Outer membrane vesicle (class 1)
PA4035	hypothetical protein	35.9	6.27	-0.245	Periplasmic (class 1)
PA4176	ppiC2; peptidyl-prolyl cis-trans isomerase	10	6.24	-0.291	Cytoplasmic (class 1,3)
PA4370	icmP; Insulin-cleaving metalloproteinase	47.2	4.45	-0.291	Outer membrane vesicle (class 1)
PA4376	pncB2; nicotinate phosphoribosyl-transferase	45.7	6.65	-0.113	Cytoplasmic (class 3)
PA4443	cysD; sulfate adenylate transferase subunit 2	35.5	7.09	-0.551	Cytoplasmic (class 1,3)
PA4450	murA; enoylpyruvate transferase	44.6	5.49	0.152	Cytoplasmic (class 3)
PA4561	ribF; riboflavin kinase	34.3	9.06	-0.081	Cytoplasmic (class 3)
PA4602	glyA3; serine hydroxymethyltransferase	45.2	5.97	-0.087	Cytoplasmic (class 2,3)

PA4657	hypothetical protein	35.9	6.83	-0.167	Unknown (class 3)
PA4676	yadF; probable carbonic anhydrase	24.2	6.01	-0.149	Cytoplasmic (class 3)
PA4759	dapB; dihydrodipicolinate reductase	28.3	5.98	0.071	Cytoplasmic (class 3)
PA4778	cueR	15	7.99	-0.591	Cytoplasmic (class 1,3)
PA4848	accC; acetyl-CoA carboxylase	48.9	6.28	-0.183	Cytoplasmic (class 3)
PA4872	oxaloacetate decarboxylase	31.3	5.38	0.121	Cytoplasmic (class 3)
PA4919	pncB1; nicotinate phosphoribosyl-transferase	46.1	6.3	-0.153	Cytoplasmic (class 3)
PA5018	msrA; peptide methionine sulfoxide reductase	23.5	5	-0.243	Unknown (class 3)
PA5143	hisB; imidazoleglycerol-phosphate dehydratase	21.9	6.51	-0.194	Cytoplasmic (class 3)
PA5312	pauC; Aldehyde dehydrogenase	53.1	5.25	-0.025	Cytoplasmic (class 3)
PA5343	hypothetical protein	30.8	6.16	-0.231	Unknown (class 3)
PA5516	pdxY; pyridoxamine kinase	31.3	6.03	0.108	Cytoplasmic (class 3)

[a] Names based on nomenclature in the Pseudomonas Genome Database.
[b] The predicted molecular weight, isoelectric point, and Kyte-Doolittle hydrophobicity value are based on the predicted primary amino acid sequence of the protein, as reported in the Pseudomonas Genome Database.
[c] The predicted subcellular localization of the protein is as reported in the Pseudomonas Genome Database, where class 1 denotes an experimentally verified localization in *P. aeruginosa*, class 2 denotes an experimental verified localization of a homolog in another organism or a paralog in *P. aeruginosa*, and class 3 denotes a PSORTb-predicted localization. Underline, predicted to have protective function in the biofilm matrix.
