# Supplementary material for: A Biofilm Matrix-Associated Protease Inhibitor Protects Pseudomonas aeruginosa from Proteolytic Attack
Source: mBio. 2018 Apr 10;9(2):e00543-18. doi: 10.1128/mBio.00543-18 (PMC5893882; doi:10.1128/mBio.00543-18)
Supplement: FIG S1 [file mbo001183821sf1.pdf]

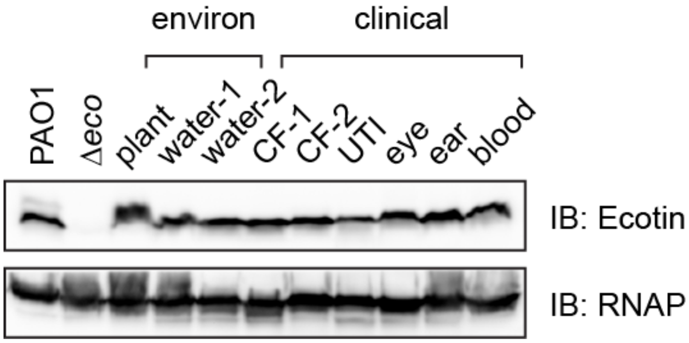

Supplementary Figure S1. Ecotin is expressed in a wide variety of *P. aeruginosa* isolates. Ecotin levels in stationary phase cells of various environmental (environ) and clinical isolates (referenced by where the strain was isolated) were determined by immunoblot (top panel). An immunoblot for RNA polymerase (RNAP, bottom panel) serves as a loading control. Our wild-type laboratory strain (PAO1) and the isogenic strain lacking ecotin (Δ*eco*) are included as a reference. Plant, strain E2; water-1, MSH3; water-2, MSH10; CF-1, CF18; CF-2, CF127; UTI, JJ692; eye, 19660; ear, T56593; blood, X13273.
