# Supplementary material for: A Biofilm Matrix-Associated Protease Inhibitor Protects Pseudomonas aeruginosa from Proteolytic Attack
Source: mBio. 2018 Apr 10;9(2):e00543-18. doi: 10.1128/mBio.00543-18 (PMC5893882; doi:10.1128/mBio.00543-18)
Supplement: FIG S2 [file mbo001183821sf2.pdf]

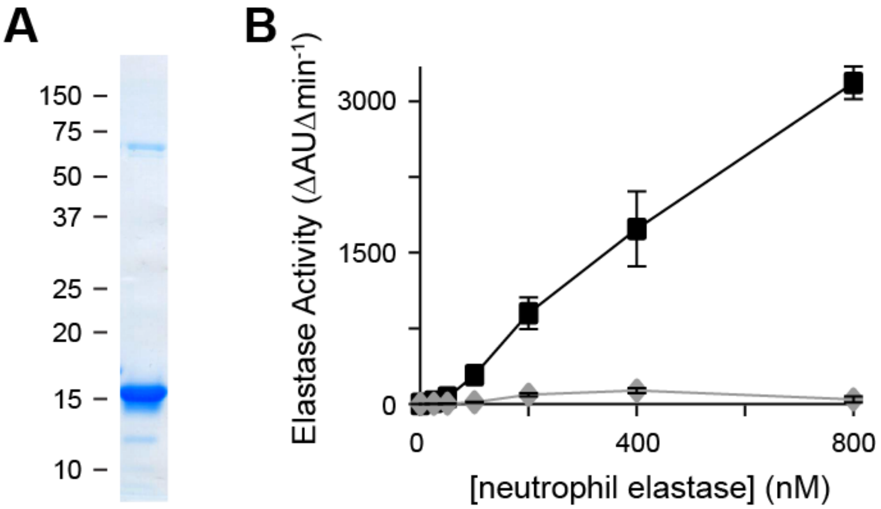

Supplementary Figure S2. Recombinant *P. aeruginosa* ecotin can inhibit neutrophil elastase *in vitro*. (A) Coomassie-stained gel of purified recombinant *P. aeruginosa* ecotin. (B) *In vitro* assay of neutrophil elastase activity using a fluorogenic substrate. Black squares, without recombinant ecotin; gray diamonds, with 2-fold molar excess of recombinant ecotin. Error bars, 1 SD.
