# Supplementary material for: A Biofilm Matrix-Associated Protease Inhibitor Protects Pseudomonas aeruginosa from Proteolytic Attack
Source: mBio. 2018 Apr 10;9(2):e00543-18. doi: 10.1128/mBio.00543-18 (PMC5893882; doi:10.1128/mBio.00543-18)
Supplement: TEXT S1 [file mbo001183821s1.pdf]

SUPPLEMENTARY METHODS

Growth conditions and antibiotics

Semi-solid media was prepared by adding 1.5% Bacto agar to LB or 1.0% noble agar to VBMM. For experiments involving induction of the *araC*-P_{BAD} promoter, L-arabinose was used at 1%. Antibiotics were used at the following concentrations: for *E. coli*, 10 mg/L gentamicin, 25 mg/L chloramphenicol, 50 mg/L kanamycin, 50 mg/L carbenicillin, and 100 mg/L ampicillin; for *P. aeruginosa*, 30 mg/L gentamicin for chromosomally integrated strains, 100 mg/L gentamicin for plasmid-borne strains, and 300 mg/L carbenicillin.

Construction of strains

Sequenced strains of *P. aeruginosa* were obtained from the Pseudomonas Genome Database (www.pseudomonas.com)(1). Bacterial genomic DNA was isolated using the DNeasy Blood & Tissue Kit (Qiagen) and PCR fragments were purified using the Qiaex II Gel Extraction Kit (Qiagen).

Using a previously described method (2), BTPa188, BTPa369, and BTPa728 were created by allelic exchange of the wild-type PAO1, WFPA801, and JJH499 strains, respectively, with pBT358. To create the *eco* deletion allele, the regions flanking *eco* were produced by PCR of genomic PAO1 DNA using the eco UpF/UpR or eco DownF/DownR primer pairs. These fragments were then connected by SOE PCR and inserted into pDONRPEX18Gm using GateWay cloning (Invitrogen) to produce pBT358.

To produce the *eco* over-expression strains, a miniTn7-compatible plasmid (pBT382) was first constructed using Multisite GateWay Technology to recombine pJJH187 and pBT363 into pUC18-miniTn7T2-Gm-GW. pBT363 was constructed by PCR of genomic PAO1 DNA with OBT469 and OBT470, followed by GateWay cloning into pDONR221 P5-P2. The over-expression construct was then integrated into the chromosome of wild-type PAO1, JJH499, and BTPa369 strains using pBT382 and pTNS2 as previously described (3).

Similar to the over-expression strains, the *eco* complementation strain was made by chromosomally inserting the allele into BTPa188 with pBT421 and pTNS2 as previously described (3). The pBT421 mini-Tn7-compatible plasmid was constructed by PCR of genomic PAO1 DNA with OBT807 and OBT810, followed by GateWay cloning into pDONRPUC18T-miniTn7T2-Gm. To enable one-step construction of miniTn7 vectors using Gateway technology, we built the plasmid pDONRPUC18T-miniTn7T2.1-Gm. A 2.5 kb DNA fragment, containing the Gateway donor site from pDONRPEX18Gm, was cloned using primers OJJH469 and OJJH470. The PCR product was then restricted with HindIII and ligated into the HindIII site of pUC18T-miniTn7T2.1-Gm using standard methods. The resulting plasmid, pDONRPUC18T-miniTn7T2.1-Gm, has the 5'-*att*P2-*ccdB*-*cat*-*att*P1-3' cassette inserted into the multiple cloning site, which was verified by Sanger sequencing using the primers OJJH1695 and OJJH1696.

To create the strain used to purify recombinant *P. aeruginosa* ecotin (BTEc314), the endogenous *eco* in *E. coli* was first deleted from BL21 (DE3) cells via Red-recombinase mediated gene replacement (4). A PCR product containing a kanamycin resistance cassette flanked by FRT sites and ~40 nucleotides of homology to the *E. coli* eco gene was created by PCR of pKD4 with OBT609 and OBT610. This product was transformed into *E. coli* BL21 (DE3) containing pKD46, and kanamycin-resistant isolates were selected. The resistance cassette was then flipped out using pCP20. The mutant was verified using OBT611 and OBT612. pBT402 was then transformed into this mutant to create BTEc314. To create pBT402, GateWay Technology was used to recombine pBT400 and p0GWA. pBT400 was constructed by PCR of

genomic PAO1 DNA with OBT616 and OBT617, followed by GateWay cloning into pDONR221 P1-P2.

Identification of proteins enriched in the matrix
Tube biofilms were grown as previously described with the following modifications (5). Cells were grown to mid-exponential phase in amine-free medium, diluted to an OD_{600} = 0.05, and injected into a 30-cm long silicone tube (6.35 mm inner diameter). After a static 1 h incubation, flow was resumed at 10 ml h^{-1} and biofilms were grown in amine-free medium for 6 days (approximately 144 h) at 25°C.

For the total biofilm proteome analysis, the biomass from 8 tube biofilms was collected in a 50-ml conical. Halt protease inhibitor (Roche) and 1 mM PMSF was added. The sample was then vortexed for 1 min, homogenized for 30 s, and bead beated for 15 min (1 min on/off cycles) with 0.1mm silica spheres (MP Biomedical) to lyse the cells. To ensure lysis of cells, viable cell counts were determined before and after this lysis step. Greater than 90% lysis was verified for both runs. The sample was then split in half. To one, 0.5 mg/ml biotinylation reagent (EZ-link sulfo-NHS-SS-biotin, Thermo-Fisher Scientific) was added, and the other was left untreated. The sample was incubated for 30 min at 25°C before the reaction was quenched for 1 h with 50 mM Tris HCl pH 7.5.

For the extracellular biofilm proteome, 0.5 mg/ml biotinylation reagent in amine-free medium was injected into four tube biofilms and incubated statically for 30m at 25°C. As a control, four tube biofilms were injected with amine-free medium lacking the biotinylation reagent. The reaction was then quenched by injecting 50mM TrisHCl pH 7.5 and incubating for 1 h. The biomass from the tubes was then collected. Halt protease inhibitor (Roche) and 1 mM PMSF was added. To remove the cells from the matrix material, the sample was then vortexed for 1 min, homogenized for 30 s, and vortexed again for 1 min with 1 ml 1mm glass beads. A similar method was previously used by Watnick and colleagues to remove the matrix from biofilm cells in *V. cholera* (6). The beads were then removed from the sample.

To purify the biotinylated proteins from the total and extracellular biofilm preparations, samples were centrifuged at 5K g for 30 min twice and passed through a 0.22 µm filter to remove cells/cell debris. The resulting supernatant (from four tubes) was incubated with 2 ml PBS-washed, high-capacity NeutrAvidin agarose beads (Thermo Fisher Scientific) for 30 min on a rotator. The beads were then packed into a column and washed with 2.5 volumes PBS with 1 mM PSMF, 25 volumes PBS with 0.5 M NaCl, 0.1% Tween-20, and 1 mM PMSF, and 6 volumes PBS. The proteins were then eluted with 50 mM DTT and then precipitated with 10% TCA. Pellets were resuspended in 0.1% RapiGest (Waters) in 50mM ammonium bicarbonate pH 7.8 and vortexed. Protein concentration was measured with a BCA assay (Thermo). Sample was alkylated with IAA (Sigma) and digested with trypsin (Promega). Samples were cleaned with MCX columns (Waters) and resuspended in 0.1% formic acid.

One µg of peptides were loaded onto an in-house packed 75-µm fused silica column (Polymicro Technologies) with one end pulled to a tip with a Sutter CO_2 laser puller packed with 25-cm C12 Jupiter (Phenomenex) 4-µm reverse-phase beads and a 100-µm fused silica Kasil (PQ Corporation) frit trap loaded with 4 cm of C12 Jupiter A Thermo Easy nanoLC was used to provide a 190 minute gradient with buffer A containing 95% water, 5% acetonitrile and 0.1% formic acid and buffer B containing 95% acetonitrile, 5% water and 0.1% formic acid at a flow rate of 250 nL/min. The effluent from the column was electrosprayed into a Thermo Velos Pro mass spectrometer. Tandem mass spectra were collected using data dependent acquisition. Two analytical replicates were run for each sample.

The tandem mass spectrometry data were searched against a *Pseudomonas aeruginosa* FASTA database from Pseudomonas Genome Database (7) including common laboratory contaminant sequences using an in house modified version of SEQUEST (8). Peptide q-values were determined via a decoy database using Percolator (9) and peptides with a q-value ≤ 0.01 were assembled into a parsimonious list of protein identifications using an in-house developed algorithm based on ID Picker (10) and integrated into MSDaPl (11). The candidate matrix proteins, which were present in two separate runs of the screen, were identified based on the following criteria: 1. had greater than or equal to two-fold spectrum counts in the extracellular biofilm proteome relative to the total biofilm proteome analysis; 2. had at least 10 spectrum counts in the extracellular biofilm proteome; and 3. had greater than or equal to two-fold spectrum counts in the biotinylated extracellular biofilm proteome relative to that without the biotinylation.

Psl co-immunoprecipitation
Psl co-immunoprecipitations were performed as previously described with the following modifications (12). Briefly, cell-free supernatants from stationary phase cultures were obtained by pelleting for 2 min at 16K g and removing the cells. PBS was then added to the supernatant to a final concentration of 1x. Anti-Psl antibodies (MedImmune) were crosslinked to magnetic Protein A Dynabeads (Life Technologies) with bis (sulfosuccinimidyl)suberate (BS^3; Thermo Fisher Scientific) according to the manufacturer's recommendations. 50 µl anti-Psl antibody-coated beads were then incubated for 30 min with 1.2 ml cell-free supernatants and then washed three times with PBS containing 0.02% Tween-20. Proteins co-precipitating with Psl were eluted with Laemmli buffer and analyzed by immunoblot.

The amount of Psl in the sample was determined as previously described (13). Briefly, cells were harvested from the stationary phase cultures and boiled with 0.5M EDTA for 20 min to release the Psl from the cells. The resulting supernatant was then treated with 0.5 mg/ml proteinase K for 1h at 60°C to degrade the proteins. The sample was then spotted on nitrocellulose and analyzed by immunoblot.

Ecotin expression profiling
For planktonic expression experiments, cells were grown at 37°C in LB broth shaking at 250 rpm and pelleted (at OD_{600} ≈ 0.2 for early exponential phase and at OD_{600} ≈ 2.0 for stationary phase). Cells were resuspended in Laemmli buffer at 10^{10} CFU/ml and analyzed by immunoblot. For the c-di-GMP experiments, 300 µg/ml carbenicillin was added to the medium to maintain the plasmid. To determine when ecotin is associated to the biofilm matrix, tube biofilms were grown as described above. At each time point, two tube biofilms were collected in a 15ml conical tube. Halt protease inhibitor (Roche) and 1 mM PMSF was added. The sample was then vortexed for 1 min, homogenized for 30 s, and vortexed again for 1 min with 1 ml 1mm glass beads. The beads were then removed from the sample. Half of the sample was then lyophilized to obtain the dry weight. To the other half of the sample, the cells were removed by pelleting at 5K g for 30 min twice. The proteins in the supernatant were then precipitated with 10% TCA and resuspended in Laemmli buffer. For SDS-PAGE, the samples were normalized based on their dry weight measurements.

For the anti-Psl immunoblot, a 250-µl aliquot of the resuspended lyophilized biofilm sample (10 mg/ml protein) was centrifuged at 13,000 *g* for 2 min, and the supernatant (containing matrix-associated Psl) was used. As positive and negative controls for Psl, supernatant fractions (matrix-associated and cell-associated) were prepared from 2-ml cultures of PAO1 and PAO1 Δ*pslD,* grown overnight at 37°C with shaking. The supernatant fractions were treated with

Proteinase K (final concentration 0.5 mg/ml) for 60 min at 60°C, followed by Proteinase K inactivation for 30 min at 80°C. The nitrocellulose membrane was loaded with 5 µl of each Proteinase K-treated supernatant fraction, and allowed to air dry for 5 min before probing.

Purification of recombinant P. aeruginosa ecotin
E. coli BL21 strain BTEc314, which has the endogenous *E. coli eco* gene deleted and carries a plasmid encoding a truncated *P. aeruginosa* ecotin (amino acids 20-157) that is tagged with the *E. coli* ecotin secretion signal peptide at the N-terminus and 10-His at the C-terminus, was used to purify the protein. Cells were induced at an OD_{600} = 0.5 with 0.5 mM IPTG and grown shaking at 18°C for 16h in 2xYT (16 g/L tryptone, 10 g/L yeast extract, 5 g/L NaCl, pH 7.0) containing ampicillin to maintain the plasmid. Cells were then harvested and lysed by sonication. The protein was purified using Nickel-NTA agarose (Bio-Rad). After elution, the purified protein was dialyzed using 20 mM Tris HCl pH 7.5 with 150 mM NaCl to remove the imidazole.

In vitro in-solution neutrophil elastase activity assay
Neutrophil elastase (Millipore), recombinant ecotin at 2-fold molar excess of the neutrophil elastase concentration, and the fluorogenic substrate (MeOSucAAPV-AMC; Millipore) at 0.25 mM were added to low salt neutrophil elastase buffer (0.1M HEPES pH 7.5, 0.1M NaCl). Cleavage of the substrate by neutrophil elastase was measured at Ex380/Em460 every 5 s for 10 mins with a Synergy Hybrid H1 Multi-mode Microplate Reader (Bio-Tek Instruments). The activity was determined as the rate of fluorescence increase.

Neutrophil elastase inhibition assay
Psl was precipitated using 50 µl anti-Psl antibody-coated Dynabeads beads from 1.2 ml cell-free supernatants buffered with PBS, as described above. The beads were then washed once in low salt neutrophil elastase buffer and resuspended in 80 µl of the buffer. Neutrophil elastase (Millipore) at 100 nM and the fluorogenic substrate (MeOSucAAPV-AMC; Millipore) at 0.25 mM was added to the beads. Cleavage of the substrate by neutrophil elastase was measured at Ex380/Em460 every 5 s for 10 mins with a Synergy Hybrid H1 Multi-mode Microplate Reader (Bio-Tek Instruments). The activity was determined as the rate of fluorescence increase. To determine the statistical significance, a Student's t-test was used. Post-experiment, the beads were recovered in PBS containing 0.02% Tween-20 and washed once. Laemmli buffer was then used to elute the protein off of the beads by boiling for 10 min, and the proteins were analyzed by SDS-PAGE as described above.

Planktonic neutrophil elastase killing assay
Cells were grown to an $OD_{600} \approx 2.0$ in tryptic soy broth (TSB; Difco) shaking at 250 rpm at 37°C. Cells were then pelleted at 1.5K g for 10 min, washed once in 10 mM sodium phosphate buffer pH 7.5 containing 1% TSB, and resuspended to 10^6 CFU/ml in the same buffer. Neutrophil elastase (Oxford Biomedical Research) at 250 µg/ml was then added to the cells and the sample was incubated rotating at 37°C for 2 h before viable cell counts were determined. The post-incubation viable cell count was subtracted from the initial cell count and $\log_{10}$ transformed to determine the log-kill. An ANOVA with post-hoc Tukey HSD test was used to determine statistical significance.

Biofilm neutrophil elastase killing assay
Stationary-phase overnight cultures were diluted 75x to seed 15-ml LB. This inoculum was placed in a 50-ml conical tube with one glass slide such that approximately half of the slide was immersed in medium. The culture was incubated 37°C without shaking for 20 h. The slide was then removed from the culture medium and gently rinsed with 3 ml 10 mM Tris pH 7.5. For samples exposed to recombinant ecotin, 100 µM ecotin was deposited over the biomass at this

point and allowed to incubate for 15 min before being rinsed with 3 ml 10 mM Tris pH 7.5. 2 μM neutrophil elastase (Millipore) was deposited over the biomass and allowed to incubate at 37°C for 30 min before being rinsed with 1 ml 10 mM Tris pH 7.5. This incubation step took place in a sealed, hydrated container to prevent the slide from drying. The adherent biomass was stained with 5 μM Syto9 (Molecular Probes) and 30 μM propidium iodide (Sigma-Aldrich) in 10 mM Tris pH 7.5 for 15 min in the dark before rinsing with 1 ml 10 mM Tris pH 7.5. The stained biomass was then covered with a coverslip and imaged on Zeiss LSM 510 confocal laser scanning microscope (Carl Zeiss). For the quantification, eight images per sample per experiment were analyzed and the experiment was conducted three independent times. Using the Volocity software (Improvision), the number of living and dead cells were identified based on the Syto9 and propidium iodide staining, respectively. The number of dead cells identified was then divided by the total number of cells identified to determine the ratio of dead cells in each image. To determine statistical significance, an ANOVA with post-hoc Tukey HSD test was used.

SUPPLEMENTARY REFERENCES

1. Winsor GL*, et al.* (2016) Enhanced annotations and features for comparing thousands of Pseudomonas genomes in the Pseudomonas genome database. *Nucleic Acids Res* 44(D1):D646-653.
2. Hmelo LR*, et al.* (2015) Precision-engineering the Pseudomonas aeruginosa genome with two-step allelic exchange. *Nat Protoc* 10(11):1820-1841.
3. Choi KH & Schweizer HP (2006) mini-Tn7 insertion in bacteria with single attTn7 sites: example Pseudomonas aeruginosa. *Nat Protoc* 1(1):153-161.
4. Datsenko KA & Wanner BL (2000) One-step inactivation of chromosomal genes in Escherichia coli K-12 using PCR products. *Proc Natl Acad Sci U S A* 97(12):6640-6645.
5. Peterson SB*, et al.* (2011) Different Methods for Culturing Biofilms In Vitro. *Biofilm Infections*, eds Bjarnsholt T, Jensen PØ, Moser C, & Høiby N (Springer, New York, NY), pp 251-266.
6. Absalon C, Van Dellen K, & Watnick PI (2011) A communal bacterial adhesin anchors biofilm and bystander cells to surfaces. *PLoS Pathog* 7(8):e1002210.
7. Winsor GL*, et al.* (2011) Pseudomonas Genome Database: improved comparative analysis and population genomics capability for Pseudomonas genomes. *Nucleic Acids Res* 39(Database issue):D596-600.
8. Eng JK, McCormack AL, & Yates JR (1994) An approach to correlate tandem mass spectral data of peptides with amino acid sequences in a protein database. *Journal of the American Society for Mass Spectrometry* 5(11):976-989.
9. Käll L, Canterbury JD, Weston J, Noble WS, & MacCoss MJ (2007) Semi-supervised learning for peptide identification from shotgun proteomics datasets. *Nature Methods* 4(11):923-925.
10. Zhang B, Chambers MC, & Tabb DL (2007) Proteomic parsimony through bipartite graph analysis improves accuracy and transparency. *J Proteome Res* 6(9):3549-3557.
11. Sharma V, Eng JK, Maccoss MJ, & Riffle M (2012) A mass spectrometry proteomics data management platform. *Mol Cell Proteomics* 11(9):824-831.
12. Borlee BR*, et al.* (2010) Pseudomonas aeruginosa uses a cyclic-di-GMP-regulated adhesin to reinforce the biofilm extracellular matrix. *Mol Microbiol* 75(4):827-842.
13. Byrd MS*, et al.* (2009) Genetic and biochemical analyses of the Pseudomonas aeruginosa Psl exopolysaccharide reveal overlapping roles for polysaccharide synthesis enzymes in Psl and LPS production. *Mol Microbiol* 73(4):622-638.
14. Simon R, Priefer U, & Pühler A (1983) A Broad Host Range Mobilization System for In Vivo Genetic Engineering: Transposon Mutagenesis in Gram Negative Bacteria. *Nat Biotechnol* 1(9):784-791.

15. Holloway BW (1955) Genetic recombination in Pseudomonas aeruginosa. *J Gen Microbiol* 13(3):572-581.
16. Zhao K*, et al.* (2013) Psl trails guide exploration and microcolony formation in Pseudomonas aeruginosa biofilms. *Nature* 497(7449):388-391.
17. Ma L, Jackson KD, Landry RM, Parsek MR, & Wozniak DJ (2006) Analysis of Pseudomonas aeruginosa conditional psl variants reveals roles for the psl polysaccharide in adhesion and maintaining biofilm structure postattachment. *J Bacteriol* 188(23):8213-8221.
18. Wolfgang MC*, et al.* (2003) Conservation of genome content and virulence determinants among clinical and environmental isolates of Pseudomonas aeruginosa. *Proc Natl Acad Sci U S A* 100(14):8484-8489.
19. Colvin KM*, et al.* (2012) The Pel and Psl polysaccharides provide Pseudomonas aeruginosa structural redundancy within the biofilm matrix. *Environ Microbiol* 14(8):1913-1928.
20. Cherepanov PP & Wackernagel W (1995) Gene disruption in Escherichia coli: TcR and KmR cassettes with the option of Flp-catalyzed excision of the antibiotic-resistance determinant. *Gene* 158(1):9-14.
21. Busso D, Delagoutte-Busso B, & Moras D (2005) Construction of a set Gateway-based destination vectors for high-throughput cloning and expression screening in Escherichia coli. *Anal Biochem* 343(2):313-321.
22. Khakimova M, Ahlgren HG, Harrison JJ, English AM, & Nguyen D (2013) The stringent response controls catalases in Pseudomonas aeruginosa and is required for hydrogen peroxide and antibiotic tolerance. *J Bacteriol* 195(9):2011-2020.
