# Supplementary material for: A Biofilm Matrix-Associated Protease Inhibitor Protects Pseudomonas aeruginosa from Proteolytic Attack
Source: mBio. 2018 Apr 10;9(2):e00543-18. doi: 10.1128/mBio.00543-18 (PMC5893882; doi:10.1128/mBio.00543-18)
Supplement: TABLE S3 [file mbo001183821st3.pdf]

Supplementary Table S3. Bacterial strains used in this study.

Strains	Relevant characteristics	Source
Escherichia coli		
NEB5α	cloning strain; *fhuA2* Δ*(argF-lacZ)U169 phoA glnV44 Φ80Δ (lacZ)M15 gyrA96 recA1 relA1 endA1 thi-1 hsdR17*	New England BioLabs
ccdB Survival™ 2 T1R	F^- *mcrA* Δ *(mrr-hsdRMS-mcrBC) Φ80lacZΔM15 ΔlacX74 recA1 araΔ139* Δ *(ara-leu)7697 galU galK rpsL endA1 nupG fhuA::IS2*, Sm^r	Invitrogen
S17.1 (λ_{pir})	conjugation donor; F^- RP4-2-Tc::Mu *aphA*::Tn7 *recA* λ_{pir} lysogen, Sm^r, Tc^r	(14)
BL21 (DE3)	expression strain; *fhuA2 [lon] ompT gal (λ DE3) [dcm]* Δ*hsdS*	New England BioLabs
BTEc314	BL21 (DE3) Δ*eco* pBT402; Ap^r	this study
Pseudomonas aeruginosa		
PAO1	Wild-type	(15)
BTPa238	PAO1 with *eco* driven by an inducible *araC*-P_{BAD} promoter integrated at attTn7 site; Gm^r	this study
JJH499	PAO1 Δ*pslD*	(16)
WFPA801	PAO1 with the promoter of *psl* replaced with *araC*-P_{BAD} promoter	(17)
BTPa188	PAO1 Δ*eco*	this study
BTPa284	JJH499 with *eco* driven by an inducible *araC*-P_{BAD} promoter integrated at attTn7 site; Gm^r	this study
BTPa369	WFPA801 Δ*eco*	this study
BTPa446	BTPa369 with *eco* driven by an inducible *araC*-P_{BAD} promoter integrated at attTn7 site; Gm^r	this study
BTPa512	BTPa188 with *eco* driven by its native promoter integrated at the attTn7 site; Gm^r	this study
BTPa728	JJH499 Δ*eco*	this study
E2	Environmental isolate from a tomato plant (Gainesville, FL)	(18)
MSH3	Environmental isolate from water (Spirit Lake, WA)	(18)
MSH10	Environmental isolate from water (Spirit Lake, WA)	(18)
CF18	Clinical isolate from a cystic fibrosis patient (Jackson, MS)	(18)
CF127	Clinical isolate from a cystic fibrosis patient (Denver, CO)	(18)
JJ692	Clinical isolate from a UTI (Minneapolis, MN)	(18)
19660	Clinical isolate from a cornea/ocular infection (Berkeley, CA)	(18)
T56593	Clinical isolate from an ear infection (Winston-Salem, NC)	(19)
X13273	Clinical isolate from a blood infection (Seattle, WA)	(18)
