# Supplementary material for: A Biofilm Matrix-Associated Protease Inhibitor Protects Pseudomonas aeruginosa from Proteolytic Attack
Source: mBio. 2018 Apr 10;9(2):e00543-18. doi: 10.1128/mBio.00543-18 (PMC5893882; doi:10.1128/mBio.00543-18)
Supplement: TABLE S4 [file mbo001183821st4.pdf]

Supplementary Table S4. Plasmids used in this study.

Plasmids	Relevant characteristics	Source
pDONRPEX18Gm	pEX18-based, GateWay-compatible suicide vector (Accession No. KM880128) with attP1 and attP2 recombination sites and *ccdB*; Gm^r, Cm^r	(2)
pUC18T-miniTn7T2-Gm-GW	GateWay-compatible miniTn7 vector with attR1 and attR2 recombination sites and *ccdB*; Ap^r, Gm^r, Cm^r	(16)
pUC18T-miniTn7T-Gm	cloning vector for gene insertion in Gm^S bacteria; Ap^r, Gm^r	(3)
pUC18T-miniTn7T2.1-Gm	pUC18T-miniTn7T-Gm with transcriptional terminators at both ends of the Tn7 transposon; Ap^r, Gm^r	this study
pDONRPUC18T-miniTn7T2-Gm	pUC18T-miniTn7T2.1-Gm-based GateWay-compatible miniTn7 vector with attP1 and attP2 recombination sites and *ccdB*; Ap^r, Gm^r, Cm^r	this study
pDONR221 P1-P2	GateWay-compatible vector with attP1 and attP2 recombination sites and *ccdB*; Km^r, Cm^r	Invitrogen
pDONR221 P5-P2	GateWay-compatible vector with attP5 and attP2 recombination sites and *ccdB*; Km^r, Cm^r	Invitrogen
pTNS2	T7 transposase expression vector; Ap^r	(3)
pKD4	Template for FRT-flanked Kn^r gene	(4)
pKD46	Plasmid that expresses the Red recombinase system; Ap^r	(4)
pCP20	Plasmid that expresses the Flp recombinase	(20)
p0GWA	GateWay-compatible expression vector	(21)
pJJH187	GateWay-compatible plasmid containing the *araC* repressor and the P_{BAD} promoter flanked by attL1 and attR5 recombination sites; Km^r	(22)
pBT358	pDONRPEX18Gm with an in-frame *eco* deletion allele; Gm^r	this study
pBT363	GateWay-compatible plasmid containing *eco* flanked by attL5 and attL2 recombination sites; Km^r	this study
pBT382	miniTn7 transposon with *eco* driven by the inducible *araC*-P_{BAD} promoter; Gm^r	this study
pBT400	GateWay-compatible plasmid containing a modified *eco* gene flanked by attL1 and attL2 recombination sites; Km^r	this study
pBT402	p0GWA-based expression plasmid to produce a recombinant *P. aeruginosa* ecotin that lacks its first 19 amino acids, has an N-terminal secretion signal peptide from *E. coli* ecotin, and has a C-terminal 10-His tag; Ap^r	this study
pBT421	miniTn7 transposon with *eco* driven by its native promoter; Gm^r	this study
