# Supplementary material for: A Biofilm Matrix-Associated Protease Inhibitor Protects Pseudomonas aeruginosa from Proteolytic Attack
Source: mBio. 2018 Apr 10;9(2):e00543-18. doi: 10.1128/mBio.00543-18 (PMC5893882; doi:10.1128/mBio.00543-18)
Supplement: TABLE S5 [file mbo001183821st5.pdf]

Supplementary Table S5. Oligonucleotides used in this study.

Primer	Sequence
OBT457 (eco UpF)	GGGGACAAGTTTGTACAAAAAAGCAGGCTCAGGACGACATCGTCGCCTTTCTC
OBT458 (eco UpR)	CGTTTATTCGCTGACCGCTTTCTCGATCAGTAGTGCTTTCATGTCCTTGAG
OBT459 (eco DownF)	GAGAAAGCGGTCAGCGAATAAACG
OBT460 (eco DownR)	GGGGACCACTTTGTACAAGAAAGCTGGGTAGACCGCAAGCCATGAAGGTCTG
OBT461 (eco SeqF)	GAGGAACCGTCATGGACACCAG
OBT462 (eco SeqR)	CAAATCGCCTACCTTAGACGGAC
OBT469 (attB5 primer for eco)	GGGGACAACTTTGTATACAAAAGTTGCGAAAGAGGAGAAATTAAGCATGAAAGCACTACTGATCGCCGC
OBT470 (attB2 primer for eco)	GGGGACCACTTTGTACAAGAAAGCTGGGTATTATTCGCTGACCGCTTTCTCGAC
OBT609 (E. coli eco primer)	AAATGAAGAAGGAAAGCAAAAAAATGAAGACCATTCTACCGTGTAGGCTGGAGCTGCTTC
OBT610 (E. coli eco primer)	TTCTCTTCCGCCTTCCAGACGCGGTACTTCACATCTACATCATATGAATATCCTCCTTAG
OBT611 (E. coli eco SeqF)	CGGGCTTTTGCTCACATTTCAAATG
OBT612 (E. coli eco SeqR)	AACCGGCTTGGGCGTTAGATG
OBT616 (attB1 primer for recombinant eco)	GGGGACAAGTTTGTACAAAAAAGCAGGCTCAATGAAGACCATTCTACCTGCAGTATTGTTTGCCGCTTTCGCTACCACTTCCGCCTGGGCGGCCAAACTGGATGAAAAGGTTCCCTAC
OBT617 (attB2 primer for recombinant eco)	GGGGACCACTTTGTACAAGAAAGCTGGGTATTATTAGTGGTGGTGATGGTGATGATGGTGGTGATGGCTGCTGCCTTCGCTGACCGCTTTCTCGAC
OBT807 (attB2 primer for eco)	GGGGACCACTTTGTACAAGAAAGCTGGGTACGTTTATTCGCTGACCGCTTTCTC
OBT810 (attB1 primer for eco)	GGGGACAAGTTTGTACAAAAAAGCAGGCTCAACCGAGGAATACGTCGAGGACCATC
M13F (-21)	GTAAAACGACGGCCAG
M13R	CAGGAAACAGCTATGAC
OJJH506 (pUC18backboneR01)	GGCCGATTCATTAATGCAGC
OJJH507 (Tn7LF01)	GTGTAAAGCCTGGGGTGC
OJJH1695 (miniTn7F01-Seq)	CTAATTCGATCATGCATGAGCTC
OJJH1696 (miniTn7R02-Seq)	CACTTATCTGGTTGGCCTGCAAG
OJJH469 (attP1-GWcassette201-F02)	ATCCGGAAGCTTGTTAACGCTAGCATGGATCTC
OJJH470 (attP2-GWcassette201-R02)	ATCCGGAAGCTTTCATGATGATATATTTTTATCTTGTGC
OJJH510 (T4-Terminator- kpnI-1)	\5Phos\-CTTGGGGACCCTAGAGGTCCCCTTTTTTTATTTTTTGGGGACCCTAGAGGTCCCCTTTTTTTATTTTGGTAC
OJJH511 (T4-Terminator- kpnI-2)	\5Phos\-CAAAATAAAAAAGGGGACCTCTAGGGTCCCCAAAAAATAAAAAAAGGGGACCTCTAGGGTCCCCAAGGTAC
